# Supplementary material for: Prevalence of Opioid Use Disorder among Patients with Cancer-Related Pain: A Systematic Review
Source: J Clin Med. 2022 Mar 14;11(6):1594. doi: 10.3390/jcm11061594 (PMC8954099; doi:10.3390/jcm11061594)
Supplement: Supplementary file 1 [file jcm-11-01594-s001.zip › jcm-1592744-supplementary.pdf]

PRISMA 2020 Checklist

Table S1. PRISMA.

Section and Topic	Item #	Checklist item	Location where item is reported
**TITLE**			
Title	1	Identify the report as a systematic review.	Line 2-3
**ABSTRACT**			
Abstract	2	See the PRISMA 2020 for Abstracts checklist.	
**INTRODUCTION**			
Rationale	3	Describe the rationale for the review in the context of existing knowledge.	Line 37-47
Objectives	4	Provide an explicit statement of the objective(s) or question(s) the review addresses.	Line 48-50
**METHODS**			
Eligibility criteria	5	Specify the inclusion and exclusion criteria for the review and how studies were grouped for the syntheses.	Line 53-54<br>Line 57-67
Information sources	6	Specify all databases, registers, websites, organisations, reference lists and other sources searched or consulted to identify studies. Specify the date when each source was last searched or consulted.	Line 57
Search strategy	7	Present the full search strategies for all databases, registers and websites, including any filters and limits used.	Line 57-67
Selection process	8	Specify the methods used to decide whether a study met the inclusion criteria of the review, including how many reviewers screened each record and each report retrieved, whether they worked independently, and if applicable, details of automation tools used in the process.	Line 68-73
Data collection process	9	Specify the methods used to collect data from reports, including how many reviewers collected data from each report, whether they worked independently, any processes for obtaining or confirming data from study investigators, and if applicable, details of automation tools used in the process.	Line 75-87
Data items	10a	List and define all outcomes for which data were sought. Specify whether all results that were compatible with each outcome domain in each study were sought (e.g. for all measures, time points, analyses), and if not, the methods used to decide which results to collect.	Line 76-77
	10b	List and define all other variables for which data were sought (e.g. participant and intervention characteristics, funding sources). Describe any assumptions made about any missing or unclear information.	Line 77-81
Study risk of bias assessment	11	Specify the methods used to assess risk of bias in the included studies, including details of the tool(s) used, how many reviewers assessed each study and whether they worked independently, and if applicable, details of automation tools used in the process.	Line 89-94
Effect measures	12	Specify for each outcome the effect measure(s) (e.g. risk ratio, mean difference) used in the synthesis or presentation of results.	Table 1
Synthesis methods	13a	Describe the processes used to decide which studies were eligible for each synthesis (e.g. tabulating the study intervention characteristics and comparing against the planned groups for each synthesis (item #5)).	Line 96-110
	13b	Describe any methods required to prepare the data for presentation or synthesis, such as handling of missing summary statistics, or data conversions.	Line 96-110
	13c	Describe any methods used to tabulate or visually display results of individual studies and syntheses.	Line 96-110
	13d	Describe any methods used to synthesize results and provide a rationale for the choice(s). If meta-analysis was performed, describe the model(s), method(s) to identify the presence and extent of statistical heterogeneity, and software package(s) used.	Line 96-115
	13e	Describe any methods used to explore possible causes of heterogeneity among study results (e.g. subgroup analysis, meta-regression).	Line 96-115
	13f	Describe any sensitivity analyses conducted to assess robustness of the synthesized results.	Line 96-110
Reporting bias assessment	14	Describe any methods used to assess risk of bias due to missing results in a synthesis (arising from reporting biases).	Line 96-110
Certainty	15	Describe any methods used to assess certainty (or confidence) in the body of evidence for an outcome.	Line 96-110

PRISMA 2020 Checklist

Section and Topic	Item #	Checklist item	Location where item is reported
assessment			
**RESULTS**			
Study selection	16a	Describe the results of the search and selection process, from the number of records identified in the search to the number of studies included in the review, ideally using a flow diagram.	Figure 1
	16b	Cite studies that might appear to meet the inclusion criteria, but which were excluded, and explain why they were excluded.	Figure 1
Study characteristics	17	Cite each included study and present its characteristics.	Table 1
Risk of bias in studies	18	Present assessments of risk of bias for each included study.	Table 1
Results of individual studies	19	For all outcomes, present, for each study: (a) summary statistics for each group (where appropriate) and (b) an effect estimate and its precision (e.g. confidence/credible interval), ideally using structured tables or plots.	Figure 2-6
Results of syntheses	20a	For each synthesis, briefly summarise the characteristics and risk of bias among contributing studies.	Table 1
	20b	Present results of all statistical syntheses conducted. If meta-analysis was done, present for each the summary estimate and its precision (e.g. confidence/credible interval) and measures of statistical heterogeneity. If comparing groups, describe the direction of the effect.	Figure 2-6
	20c	Present results of all investigations of possible causes of heterogeneity among study results.	Figure 4
	20d	Present results of all sensitivity analyses conducted to assess the robustness of the synthesized results.	n.a.
Reporting biases	21	Present assessments of risk of bias due to missing results (arising from reporting biases) for each synthesis assessed.	I^2 in Figure 2-6
Certainty of evidence	22	Present assessments of certainty (or confidence) in the body of evidence for each outcome assessed.	Figure 2-6
**DISCUSSION**			
Discussion	23a	Provide a general interpretation of the results in the context of other evidence.	Line 206-217
	23b	Discuss any limitations of the evidence included in the review.	Line 184-202
	23c	Discuss any limitations of the review processes used.	Line 167-183
	23d	Discuss implications of the results for practice, policy, and future research.	Line 219-225
**OTHER INFORMATION**			
Registration and protocol	24a	Provide registration information for the review, including register name and registration number, or state that the review was not registered.	Line 54-55
	24b	Indicate where the review protocol can be accessed, or state that a protocol was not prepared.	Line 54-55
	24c	Describe and explain any amendments to information provided at registration or in the protocol.	Line 54-55
Support	25	Describe sources of financial or non-financial support for the review, and the role of the funders or sponsors in the review.	Line 235
Competing interests	26	Declare any competing interests of review authors.	Line 238
Availability of data, code and other materials	27	Report which of the following are publicly available and where they can be found: template data collection forms; data extracted from included studies; data used for all analyses; analytic code; any other materials used in the review.	Line 228-229

PRISMA 2020 for Abstracts Checklist

Section and Topic	Item #	Checklist item	Reported (Yes/No)
**TITLE**			
Title	1	Identify the report as a systematic review.	Yes
**BACKGROUND**			
Objectives	2	Provide an explicit statement of the main objective(s) or question(s) the review addresses.	Yes
**METHODS**			
Eligibility criteria	3	Specify the inclusion and exclusion criteria for the review.	No
Information sources	4	Specify the information sources (e.g. databases, registers) used to identify studies and the date when each was last searched.	Yes
Risk of bias	5	Specify the methods used to assess risk of bias in the included studies.	No
Synthesis of results	6	Specify the methods used to present and synthesise results.	Yes
**RESULTS**			
Included studies	7	Give the total number of included studies and participants and summarise relevant characteristics of studies.	No
Synthesis of results	8	Present results for main outcomes, preferably indicating the number of included studies and participants for each. If meta-analysis was done, report the summary estimate and confidence/credible interval. If comparing groups, indicate the direction of the effect (i.e. which group is favoured).	Yes
**DISCUSSION**			
Limitations of evidence	9	Provide a brief summary of the limitations of the evidence included in the review (e.g. study risk of bias, inconsistency and imprecision).	No
Interpretation	10	Provide a general interpretation of the results and important implications.	Yes
**OTHER**			
Funding	11	Specify the primary source of funding for the review.	No
Registration	12	Provide the register name and registration number.	No

From [36]: Page MJ, McKenzie JE, Bossuyt PM, Boutron I, Hoffmann TC, Mulrow CD, et al. The PRISMA 2020 statement: an updated guideline for reporting systematic reviews. BMJ 2021;372:n71. doi: 10.1136/bmj.n71

For more information, visit: http://www.prisma-statement.org/

Table S2. Search equation.

Equation Cochrane
#1 "opioids":ti,ab,kw OR (analgesic* OR agonist*):ti,ab,kw NEAR opioid:ti,ab,kw #2 ("neoplasms" OR "neoplasia" OR "tumor" OR "Neoplasias" OR "Malignancy" OR "Malignancies" OR "cancer" OR "tumors" OR "Cancers" OR "neoplasm" OR "carcinoma" OR oncology* OR "Tumour" OR "Neoplastic growth" OR "New growth" OR "Neoplastic disease"):ti,ab,kw NEAR ("soreness" OR "backache" OR "aches" OR "pain" OR "physical suffering" OR "back ache" OR "painful" OR "physical sufferings" OR "aching" OR "ache" OR "pains" OR "dolor" OR "suffering, physical"):ti,ab,kw #3 ("misuse" OR "non-medical" OR "nmupd" OR "overdoses" OR "overdose" OR "abuse" OR "addition" OR aberrant):ti,ab,kw #4 ("non-cancer" OR noncancer OR non-malignant OR Non-opioid* OR non-cancerous):ti #5 #1 AND #2 AND #3 NOT 4
**Equation Embase**
(('pain'/exp OR 'pain measurement'/exp OR 'pain clinic'/exp OR 'nociception'/exp OR 'pain assessment'/exp OR 'pain control'/exp) AND 'neoplasm'/exp OR 'cancer pain'/exp OR 'tumor pain'/exp) AND 'opiate agonist'/exp NOT (noncancer*:ti OR 'non-cancer*':ti OR nonmalignant:ti OR 'nonmalignant*':ti OR 'non-opioid*':ti OR 'non-opiate':ti) AND ('drug misuse'/exp OR 'substance abuse'/exp OR 'drug overdose'/exp OR 'drug misuse':ti,ab,kw OR 'non-medical use':ti,ab,kw OR nmupd:ti,ab,kw) AND [embase]/lim NOT ([embase]/lim AND [medline]/lim)
**Equation Pubmed**
(((("pain"[MeSH Terms] OR "pain measurement"[MeSH Terms] OR "pain clinics"[MeSH Terms] OR "pain perception"[MeSH Terms] OR "pain management"[MeSH Terms]) AND "analgesics, opioid"[MeSH Terms] AND "neoplasms"[MeSH Terms]) OR ("Cancer Pain"[MH] AND "analgesics, opioid"[MeSH Terms])) OR ((((("opioids"[TW] OR "analgesics, opioid"[TW] OR "full opioid agonists"[TW] OR "opioid mixed agonist antagonists"[TW] OR "opioid full agonists"[TW] OR "opioid analgesics"[TW] OR "opioid agonists, partial"[TW] OR "opioid partial agonists"[TW] OR "opioid mixed agonist-antagonists"[TW] OR "partial opioid agonists"[TW]) AND (("neoplasms"[TW] OR "Malignant Neoplasms"[TW] OR "Benign Neoplasm"[TW] OR "neoplasia"[TW] OR "tumor"[TW] OR "Malignant Neoplasm"[TW] OR "Neoplasias"[TW] OR "NEOPLASM MALIGNANT"[TW] OR "neoplasm benign"[TW] OR "Malignancy"[TW] OR "Malignancies"[TW] OR "neoplasms benign"[TW] OR "cancer"[TW] OR "benign neoplasms"[TW] OR "tumors"[TW] OR "neoplasms malignant"[TW] OR "Cancers"[TW] OR "neoplasm"[TW] OR "carcinoma"[TW] OR "oncology"[TW] OR "NEOPLASM MALIGNANT"[TW] OR "cancer nos"[TW] OR "NEOPLASM NOS"[TW] OR "Malignant tumor"[TW] OR "ca cancer"[TW] OR "Malignant tumour"[TW] OR "Malignant neoplastic disease"[TW] OR "Malignant neoplasm NOS"[TW] OR "Tumour"[TW] OR "Neoplastic growth"[TW] OR "New growth"[TW] OR "Neoplastic disease"[TW] OR "risk factor for malignancy"[TW]))) AND (("pain"[MH] OR "soreness"[TW] OR "backache"[TW] OR "aches"[TW] OR "pain"[TW] OR "physical suffering"[TW] OR "back ache"[TW] OR "painful"[TW] OR "physical sufferings"[TW] OR "aching"[TW] OR "ache"[TW] OR "pains"[TW] OR "dolor"[TW] OR "suffering, physical"[TW]))) NOT (("non-cancer"[Title] OR noncancer[Title] OR non-malignant[Title] OR Non-opioid*[Title] OR non-cancerous[Title]))) OR ((("opioids"[TW] OR "analgesics, opioid"[TW] OR "full opioid agonists"[TW] OR "opioid mixed agonist antagonists"[TW] OR "opioid full agonists"[TW] OR "opioid analgesics"[TW] OR "opioid agonists, partial"[TW] OR "opioid partial agonists"[TW] OR "opioid mixed agonist-antagonists"[TW] OR "partial opioid agonists"[TW]) AND (("tumor-related pain"[TW] OR "cancer pains"[TW] OR "cancer-associated pain"[TW] OR "cancer related pain"[TW] OR "cancer pain"[TW] OR "cancer-related pain"[TW] OR "pain, cancer"[TW] OR "Cancer Pain"[TW] OR "neoplasm related pain"[TW] OR "cancer associated pain"[TW] OR "pain cancer"[TW] OR "neoplasm-related pain"[TW] OR "tumor-associated pain"[TW] OR "oncological pain"[TW] OR "tumor associated pain"[TW] OR "tumor related pain"[TW] OR "oncology pain"[TW] OR "cancer-related pains"[TW] OR "oncological pains"[TW] OR (cancer*[Title] OR neoplasm*[Title] AND

pain*[Title])))) NOT (("noncancer"[Title] OR noncancer[Title] OR non-malignant[Title] OR Non-opioid*[Title] OR noncancerous[Title]))))) AND ("Drug Misuse"[MH] OR "misuse"[TW] OR "non-medical"[TW] OR "nmupd"[TW] OR "drug overdose"[MH] OR "overdoses"[TW] OR "overdose"[TW] OR "Substance Abuse, Oral"[MH] OR "abuse"[TW] OR "addition"[TW] OR aberrant[TW])
